# Degradation Potential of Protocatechuate 3,4-Dioxygenase from Crude Extract of *Stenotrophomonas maltophilia* Strain KB2 Immobilized in Calcium Alginate Hydrogels and on Glyoxyl Agarose

**DOI:** 10.1155/2014/138768

**Published:** 2014-02-12

**Authors:** Urszula Guzik, Katarzyna Hupert-Kocurek, Marta Krysiak, Danuta Wojcieszyńska

**Affiliations:** Department of Biochemistry, Faculty of Biology and Environmental Protection, University of Silesia in Katowice, Jagiellonska 28, 40-032 Katowice, Poland

## Abstract

Microbial intradiol dioxygenases have been shown to have a great potential for bioremediation; however, their structure is sensitive to various environmental and chemical agents. Immobilization techniques allow for the improvement of enzyme properties. This is the first report on use of glyoxyl agarose and calcium alginate as matrixes for the immobilization of protocatechuate 3,4-dioxygenase. Multipoint attachment of the enzyme to the carrier caused maintenance of its initial activity during the 21 days. Immobilization of dioxygenase in calcium alginate or on glyoxyl agarose resulted in decrease in the optimum temperature by 5°C and 10°C, respectively. Entrapment of the enzyme in alginate gel shifted its optimum pH towards high-alkaline pH while immobilization of the enzyme on glyoxyl agarose did not influence pH profile of the enzyme. Protocatechuate 3,4-dioygenase immobilized in calcium alginate showed increased activity towards 2,5-dihydroxybenzoate, caffeic acid, 2,3-dihydroxybenzoate, and 3,5-dihydroxybenzoate. Slightly lower activity of the enzyme was observed after its immobilization on glyoxyl agarose. Entrapment of the enzyme in alginate gel protected it against chelators and aliphatic alcohols while its immobilization on glyoxyl agarose enhanced enzyme resistance to inactivation by metal ions.

## 1. Introduction

Protocatechuate 3,4-dioxygenase belongs to the iron-dependent enzymes and catalyzes intradiol cleavage of aromatic compounds [1]. The active site of this enzyme contains two tyrosines, two histidines, and a hydroxide ion as ligands of the high-spin iron(III) [2]. Interactions between substrate and atom of iron(III) cause activation of the substrate for an electrophilic attack by molecular oxygen. It leads to the peroxobridge formation between the iron and C4 of substrate. Next, Criegee rearrangement of this structure occurs, leading to the cyclic anhydride formation [1]. These enzymes take part in degradation of various aromatic compounds but they can be inhibited by different agents such as substrate analogues, chelators, and metal ions [3–7]. For that reason, identification and isolation of enzymes resistant to these factors have become an important subject of the research and are crucial for environment bioremediation. However, direct application of enzymes in environmental treatment technologies is limited due to the loss of enzymes' activity [8]. Therefore, different methods of their stabilization have been developed. One of them is immobilization which has been used as a tool to improve many of enzymes' properties such as operational stability, inhibitor resistance, and performance in organic solvents [8–10].

Calcium alginate gel is well known and the most widely used carrier in bioremediation process [9, 11]. It is nontoxic and inexpensive. Entrapment of enzymes in calcium alginate gel protects them against environmental factors such as pH, temperature, oxygen, organic solvent, or chelators, but has the drawback of mass transfer limitation and low enzyme loading [10, 12]. Better stabilization of the enzyme can be achieved by its multipoint attachment to the carrier since formation of additional covalent bonds increases the rigidity of the immobilized enzyme [13, 14]. Among different methods of multipoint covalent strategies, one of the most effective is immobilization on glyoxyl-agarose. In this method aldehyde groups of resin react with exposed primary amino groups of the enzyme [15, 16].

Protocatechuate 3,4-dioxygenase form *Stenotrophomonas maltophilia* KB2, which catalyzes nitrophenol, is highly resistant to metal ions [6, 7]. Because of high biotransformation potential of this enzyme, in this study we have attempted to improve its functional and thermal stability as well as resistance to inhibitors through noncovalent immobilization in calcium alginate hydrogel or multipoint covalent immobilization on glyoxyl-agarose.

## 2. Materials and Methods

### 2.1. Media and Culture Conditions

*Stenotrophomonas maltophilia* KB2 (VTT E-113197) was enriched in mineral salts medium (MSM), as described previously [7], in the presence of 6 mM 4-hydroxybenzoic acid. Cultures were incubated at 30°C and agitated at 130 rpm.

### 2.2. Preparation of Cell Extracts

Cells were harvested in the late exponential growth phase (after 15 hours) and centrifuged at 4,500 g for 15 min at 4°C. Next, the cells were washed with 50 mM phosphate buffer, pH 7.0, and resuspended in the same buffer. Cells were sonicated 6x for 15 s and centrifuged at 9,000 g for 30 min at 4°C. The supernatant was used as crude extract for enzyme assays and immobilization procedures.

### 2.3. Calcium Alginate Hydrogel Formation

For immobilization of protocatechuate 3,4-dioxygenase in calcium alginate, 3 mL of enzyme solution were suspended in 7 mL of 3% (w/v) sodium alginate in 50 mM phosphate buffer solution (pH 7.0) and homogenized. After homogenization the mixture was dropped into 25 mL of 0.15 M CaCl_2_ solution. Upon contact with the solution, drops were gelled to form constant and defined-sized spheres (external diameter 2.0 mm), which remained in the solution, under mild agitation, to complete the gel formation. After 1 h of incubation, the beads were removed by vacuum filtration, washed three times with phosphate buffer solution (pH 7.0), and stored at 4°C. Such prepared alginate beads were used to analyze properties of immobilized enzyme.

### 2.4. Immobilization of Protocatechuate 3,4-Dioxygenase on Glyoxyl Agarose

Immobilization of protocatechuate 3,4-dioxygenase on glyoxyl agarose was prepared as previously described [13]. 1 mL of glyoxyl agarose was mixed with 9 mL crude extract. The progress of the immobilization was monitored by measuring enzymatic activity until the activity measurements remained constant, which indicated complete immobilization. The resulting derivatives were reduced with sodium borohydride. After 30 minutes borohydride was eliminated by washing with 50 mM phosphate buffer (pH 7.0) and abundant distilled water.

### 2.5. Enzyme Assays

9 mM 4-hydroxybenzoate was used as the inducer of protocatechuate 3,4-dioxygenase. Specific activity of free and immobilized protocatechuate 3,4-dioxygenase was assayed by measuring oxygen consumption [6]. After the addition of the enzyme (in either free or immobilized form), vials were incubated at 30°C in water-bath with shaking. At regular time intervals (30 s), the reaction progress was monitored by measurements of oxygen consumption.

One unit of enzyme activity was defined as the amount of enzyme required to generate 1 *μ*moL of product per minute at 35°C. Activity of free and immobilized enzyme was expressed as specific activity (U/mg protein). The soluble and immobilized protein concentration was determined by the dye-binding procedure of Bradford using bovine serum albumin as a standard, as described previously [10].

### 2.6. pH and Temperature Optima of Free and Immobilized Protocatechuate 3,4-Dioxygenase

The effect of pH on the enzyme activity was determined by measuring the activity at 30°C over the pH range 2.2–10.0 using the following buffers: 0.05 M phosphate-citrate (pH 4.0 to 4.5), 0.05 M Sörensen (pH 5.0), 0.05 M phosphate (pH 5.7 to 8.0), 0.05 glycine (pH 10.0), 0.05 Britton-Robinson (pH 11.00 to 12.00), and 0.05 ammonia-sodium hydroxide (pH 13.00-14.00).

The optimum temperature was determined by assaying the enzyme activity at various temperatures (4 to 65°C) in 50 mM phosphate buffer solution (pH 7.4). The enzyme and the substrate solutions were preincubated, mixed, and followed by the enzymatic reaction at the same temperature.

### 2.7. Substrate Specificity

Impact of various substituted derivatives of aromatic compounds on activity of free and immobilized enzyme was evaluated by incubating the enzyme with the respective aromatic compound for 3 min and assaying the activity. As a substrate, aromatic dihydroxy acids: 2,3-; 2,4-; 2,5-; 2,6-; 3,5-dihydroxybenzoate, caffeic acid, and 3,4-dihydroxyhydrocinnamic acid at 1 mM concentration were used.

### 2.8. Activity of Enzyme in the Presence of Inhibitors

Impact of various aliphatic alcohols and chelators on both free and immobilized enzyme was evaluated by incubating the enzyme with the respective inhibitor for 3 min and then assaying the residual activity. At regular time intervals (30 s), reaction progress was monitored by measuring oxygen consumption. Assay of catechol 2,3-dioxygenase was proceeding in the same way as in the case of noninhibited. Aliphatic alcohols studied were methanol, ethanol, propanol, and butanol at 100 mM, 200 mM, and 300 mM concentrations. For inhibition studies EDTA, 2,2′-dipyridyl, and phenanthroline at 1 mM, 2 mM, and 3 mM concentrations were used.

## 3. Results and Discussion

### 3.1. Storage Stability

**Figure 1:**
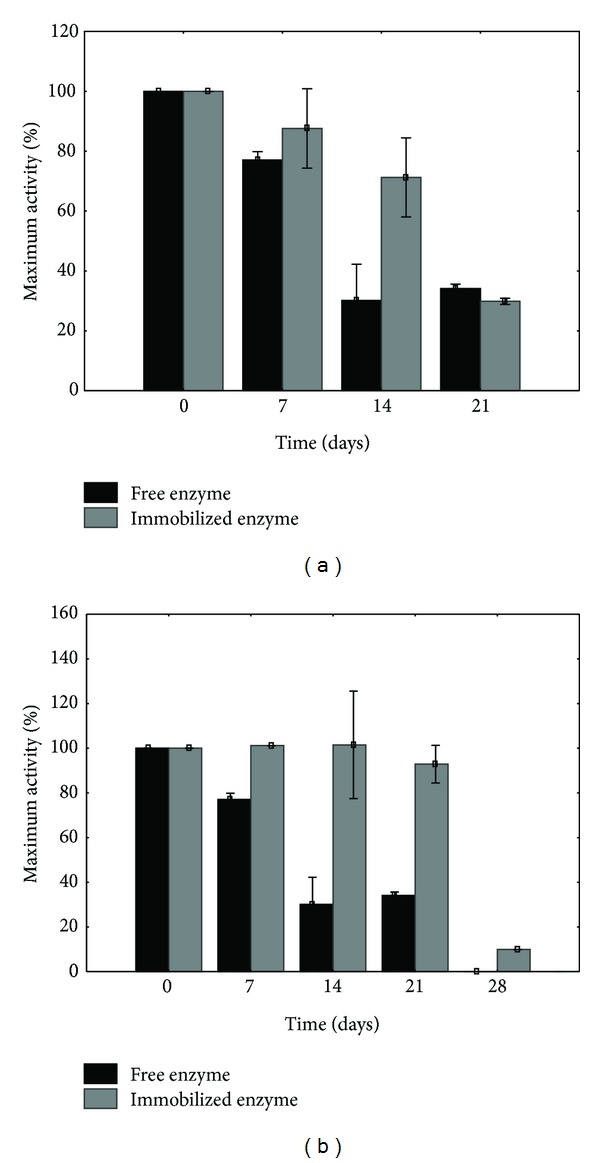
Storage stabilities of protocatechuate 3,4-dioxygenase from *Stenotrophomonas maltophilia* KB2 immobilized in calcium alginate (a) or on glyoxyl agarose (b). Data shown represent the average of three independent trials.

Protocatechuate 3,4-dioxygenase was immobilized in calcium alginate or on glyoxyl agarose. One of the most important parameters which should be considered during immobilization of enzyme is storage stability because of its practical application. The stability of free and the immobilized protocatechuate 3,4-dioxygenase was determined after storage of preparations in the phosphate buffers (50 mM, pH 7.0, for free enzyme or enzyme immobilized in calcium alginate and 40 mM, pH 8.0, for protocatechuate 3,4-dioxygenase immobilized on glyoxyl agarose) at 4°C for predetermined period. The entrapment of the protocatechuate 3,4-dioxygenase in calcium alginate matrix did not significantly influence the storage stability (Figure 1(a)). Multipoint attachment of the enzyme to the carrier (glyoxyl agarose) caused maintenance of its initial activity for a period of 21 days (Figure 1(b)). Moreover, immobilized enzyme still showed 9.93% of its initial activity after 28 days of storage, while activity of the free enzyme was not observed.

### 3.2. Influence of Enzyme Immobilization on pH and Temperature Optimum

**Figure 2:**
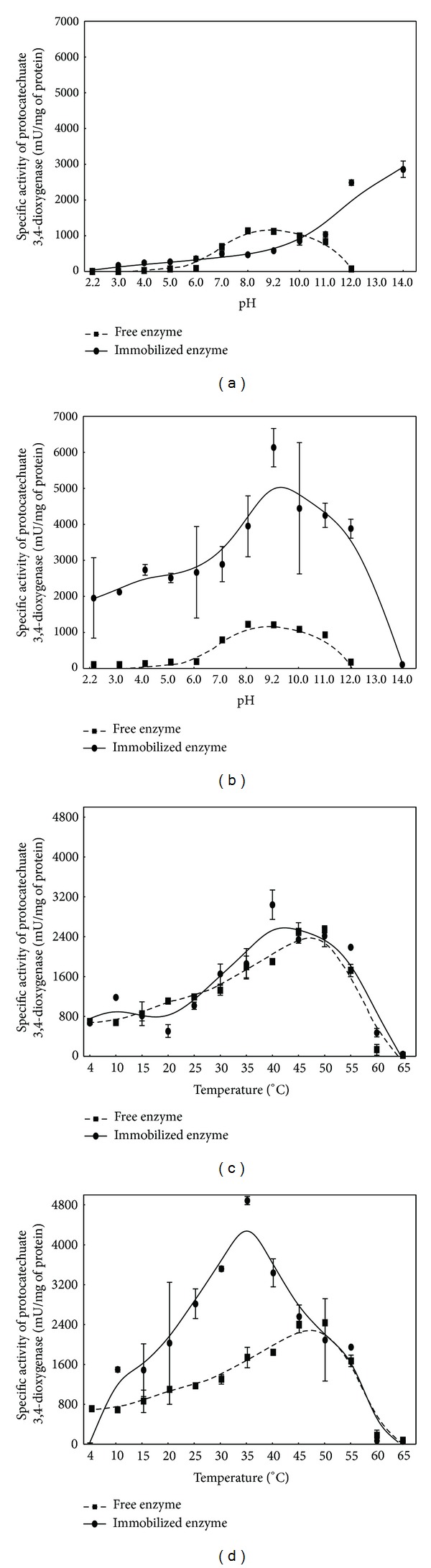
Effects of pH ((a), (b)) and temperature ((c), (d)) on protocatechuate 3,4-dioxygenase from *Stenotrophomonas maltophilia* KB2 immobilized in calcium alginate ((a), (c)) or on glyoxyl agarose ((b), (d)). The data points represent the average of 3 independent experiments.

Environmental factors affecting enzymatic reactions include temperature and pH. Determination of the influence of protocatechuate 3,4-dioxygenase immobilization on pH optimum showed that entrapment of the enzyme in calcium alginate gel shifted its optimum pH towards high-alkaline pH (Figure 2(a)). In contrast, immobilization of the enzyme on glyoxyl agarose did not influence its pH profile. However, immobilization of protocatechuate 3,4-dioxygenase on this carrier caused significant increase of its activity (Figure 2(b)). Comparison of the temperature-activity profiles (Figures 2(c) and 2(d)) showed that the enzyme immobilized either in calcium alginate or on glyoxyl agarose had approximately 5°C and 10°C lower optimum temperature than free enzyme. Additionally, higher activity of the enzyme immobilized on glyoxyl agarose was observed (Figure 2(d)). It may result from the formation of covalent bonds between the enzyme and the carrier and the increase of enzyme rigidity [17]. Similarly, higher activity of enzyme after its immobilization was observed by Singh and Ahmed [18] and Cristovao et al. [19].

### 3.3. Effect of Immobilization on Substrate Specificity

Many of intradiol dioxygenases show narrow substrate specificity and regioselectivity [3, 20, 21]. These features make them attractive tools for biocatalytic production of fine chemical, pharmaceutical, or food ingredients. However, in bioremediation processes enzymes with wide substrate specificity are desirable.

Protocatechuate 3,4-dioxygenase from KB2 strain showed activity against various dihydroxybenzoic acids; however, its activity towards these compounds was lower than against primary substrate (protocatechuic acid). Immobilization of enzyme in both carriers increased its activity against all examined substrates (Table 1). Activity of the enzyme immobilized in calcium alginate increased particularly towards 2,5-dihydroxybenzoate, caffeic acid, 2,3-dihydroxybenzoate, and 3,5-dihydroxybenzoate. Caffeic acid and 3,4-dihydroxyhydrocinnamic acid possess the same configuration of hydroxyl group as protocatechuic acid. Immobilization of enzyme in calcium alginate increased its activity despite the presence of large substituent at 1 C position of substrate. Slightly lower activity of the enzyme was observed after its immobilization on glyoxyl agarose (Table 1). Higher activity of protocatechuate 3,4-dioxygenase toward *ortho*-diphenols with large substituents at the 4-position was observed by Hammer et al. [21]. This effect could be related to interactions between amino acid residues at the entrance into the active site and the carriers that extended area of substrate entrance. Various activities of enzyme towards tested substrates may also be connected with the type of matrix used for immobilization. Enzyme immobilized in calcium alginate gel binds to the carrier by electrostatic interaction while glyoxyl agarose is linked with the enzyme by covalent bonds. Since positively charged amino acids are localized at the entrance of crevasse, the electrostatic interaction plays the key role in modulation of enzyme activity [2, 22, 23]. Significant increase of enzyme activity after its immobilization in calcium alginate was probably caused by strong electrostatic interactions between positively charged amino acid residues of the enzyme and negatively charged groups of alginate.

Surprisingly, significant increase in activity of protocatechuate 3,4-dioxygenase immobilized in calcium alginate against 2,3-dihydroxybenzoate, 2,5-dihydroxybenzoate, and 3,5-dihydroxybenzoate (Table 1) was observed. These compounds are nontypical substrates for this enzyme because of hydroxyl group configuration. Each of the above-mentioned substrates possesses at least one hydroxyl group in *meta* position. High activity of immobilized protocatechuate 3,4-dioxygenase towards 2,3-dihydroxybenzoate, 2,5-dihydroxybenzoate, and 3,5-dihydroxybenzoate could be connected with modification of its catalytic mechanism as only 2,3-dihydroxybenzoate is in configuration typical for protocatechuate 3,4-dioxygenase's substrate. Complete change of enzyme activity after its immobilization was often observed. For example, urokinase immobilized on glyoxyl agarose showed activity of glutathione S-transferase or lipase after its immobilization in organic solvents catalysed transesterification reaction [14, 24].

### 3.4. Enzyme Activity in the Presence of Inhibitors

Inhibitors interact with specific regions of the enzyme and inhibit enzymes. For that reason immobilization may reduce inhibition in two different ways based on the type of inhibition. After immobilization allosteric site of enzyme may be blocked which makes interaction between this site and allosteric inhibitor impossible. Immobilization may also slightly deform enzyme structure and as a consequence influence changes in affinity of inhibitor to the active site of enzyme [14].

Protocatechuate 3,4-dioxygenase possesses an iron ion in the active site and therefore it was interesting to check the influence of aliphatic alcohols on enzyme activity after its immobilization. As it is known, aliphatic alcohols may coordinate metal ions and have an influence on the reaction microenvironment [1, 25, 26]. After immobilization of enzyme in calcium alginate we did not observe its inhibition by aliphatic alcohols at all tested concentration (Table 2). Only 0.3 mM methanol weakly inhibited examined enzyme. This effect is connected with small size of methanol molecule, which penetrates the extended entrance into the active site. Strong inhibition of enzymatic activity by aliphatic alcohols, especially methanol and ethanol, was observed after immobilization of protocatechuate 3,4-dioxygenase on glyoxyl agarose. Low molecular alcohols inhibited the examined enzyme after immobilization on glyoxyl agarose due to its strong hydrogen interaction with the enzyme in this environment [10, 26]. Changes in enzyme structure after its immobilization caused by covalent interaction made the enzyme more sensitive to aliphatic alcohols.

Protective effect of immobilization in calcium alginate on enzyme activity was observed in the presence of Cd^2+^, Al^3+^, and Fe^3+^ while in the presence of Mn^2+^ and Zn^2+^ inhibitory effect of metals on enzyme activity increased (Table 3). Protocatechuate 3,4-dioxygenase activation in the presence of Fe^3+^ may be caused by greater availability of this ion for the active site. Untypical high activity of the enzyme in the presence of Al^3+^ after entrapment of enzyme requires further research.

Immobilization of enzyme on glyoxyl agarose showed better protective effect than immobilization in calcium alginate (Table 3). Only in the presence of Ni^2+^ the inhibition increased. Nies [27] suggested that toxicity of Ni^2+^ is connected with tendency of this cation to bind to the cysteine or histidine residues of the protein. In the active site of intradiol dioxygenases, the iron is coordinated by two tyrosines and two histidines [1, 2]. Probably, the interaction between these residues and Ni^2+^ decreased enzyme activity after its immobilization [6, 27]. Cu^2+^, Cd^2+^, Al^3+^, and Fe^3+^ showed weaker effect on the enzyme activity (Table 3). Gopal et al. [28] demonstrated that ions of the same charge as the ions in the active site can replace them and in this way modulate enzyme activity. Since in the active site of protocatechuate 3,4-dioxygenase from KB2 Fe^3+^ ion is located, it could be replaced by Al^3+^. However, covalent immobilization of the enzyme increases its structure rigidity that makes the exchange of ions impossible. In our previous work we suggested that binding of transition metal ions such as Cu^2+^, Cd^2+^, Ni^2+^, Mn^2+^, or Zn^2+^ might deactivate enzyme by their interaction with the thiol groups of protocatechuate 3,4-dioxygenase [6]. The weaker inhibitory effect of Cu^2+^ and Cd^2+^ is connected with limited mobility of protein chain after its immobilization.

It is known that enzymes possessing metal ions at their active sites are sensitive to chelators [4, 29, 30]. Despite this, after immobilization of protocatechuate 3,4-dioxygenase from KB2 strain in calcium alginate gel, we did not observe inhibition of the enzyme in the presence of ferrous or ferric chelators (Table 4). Varga and Neujahr [31] observed correlation between substrate specificity and the strength of iron binding. However, our results show an opposite trend. After immobilization in calcium alginate gel protocatechuate 3,4-dioxygenase showed wide substrate specificity and the iron was strongly bind at the active site. In contrast protocatechuate 3,4-dioxygenases immobilized on glyoxyl agarose was inhibited by chelators at all tested concentrations (Table 4). Differences in the enzymatic behavior of protocatechuate 3,4-dioxygenases depending on the method of immobilization indicate various interactions between protein and the carrier.

## 4. Conclusions

Immobilization of protocatechuate 3,4-dioxygenase from *Stenotrophomonas maltophilia* KB2 has been shown to be an effective method for improving the substrate specificity of the enzyme. Differences in the enzyme behavior after immobilization in calcium alginate or on glyoxyl agarose resulted from the various types of interactions between the enzyme and the carrier. Entrapment of protocatechuate 3,4-dioxygenase protected the enzyme against chelators and aliphatic alcohols while covalent binding of the enzyme to the carrier caused its higher stability in the presence of metal ions. It should be emphasized that the differences in properties of the enzyme immobilized on glyoxyl agarose and in alginate may contribute to wider use of this enzyme in bioremediation. Production of biopreparat which contains the enzyme immobilized in different matrix seems to be the best way to improve degradation potential of protocatechuate 3,4-dioxygenase.

## Figures and Tables

**Table 1:** Substrate specificity of immobilized protocatechuate 3,4-dioxygenase from *Stenotrophomonas maltophilia* KB2. Data shown represent the average of three independent trials ± standard deviation.

Substrate	Relative activity of free enzyme, %	Relative activity of the enzyme immobilized in calcium alginate, %	Relative activity of the enzyme immobilized on glyoxyl agarose, %
Control-protocatechuate	100.0 ± 0.00	100.0 ± 0.00	100.0 ± 0.00
2,3-dihydroxybenzoate	23.60 ± 3.98	149.34 ± 22.94	119.83 ± 7.83
2,4-dihydroxybenzoate	43.50 ± 5.73	96.52 ± 16.78	65.60 ± 13.61
2,5-dihydroxybenzoate	33.46 ± 0.00	158.62 ± 11.71	72.11 ± 12.59
2,6-dihydroxybenzoate	30.51 ± 2.05	90.40 ± 6.09	36.15 ± 7.42
3,5-dihydroxybenzoate	44.33 ± 3.18	113.25 ± 2.81	42.86 ± 13.61
Caffeic acid	34.21 ± 9.65	150.00 ± 16.39	97.08 ± 11.13
3,4-dihydroxyhydrocinnamic acid	29.54 ± 5.42	95.03 ± 7.02	41.11 ± 15.26

**Table 2:** Effect of aliphatic alcohols on the activity of immobilized protocatechuate 3,4-dioxygenase from *Stenotrophomonas maltophilia* KB2. Data shown represent the average of three independent trials ± standard deviation.

Compound	Concentration, (μM)	Relative activity of free enzyme, %	Relative activity of the enzyme immobilized in calcium alginate, %	Relative activity of the enzyme immobilized on glyoxyl agarose, %
None		100.0 ± 0.00	100.0 ± 0.00	100.0 ± 0.00

Methanol	100	96.53 ± 0.54	110.70 ± 6.88	37.07 ± 17.47
	200	91.53 ± 2.18	90.08 ± 2.48	45.17 ± 9.28
	300	101.54 ± 2.18	74.71 ± 2.2	100.00 ± 13.65

Ethanol	100	144.61 ± 13.06	90.47 ± 3.58	67.18 ± 14.20
	200	156.69 ± 3.81	116.54 ± 1.92	52.51 ± 5.46
	300	141.92 ± 1.63	155.65 ± 22.01	50.58 ± 28.94

Propanol	100	160.78 ± 22.84	100.39 ± 4.40	93.82 ± 9.28
	200	134.23 ± 5.98	101.75 ± 7.98	78.38 ± 8.19
	300	130.38 ± 1.63	101.75 ± 4.13	76.83 ± 11.47

Butanol	100	112.69 ± 1.63	122.28 ± 0.83	108.49 ± 3.82
	200	120.77 ± 2.18	118.10 ± 11.28	86.87 ± 30.03
	300	120.38 ± 2.72	109.15 ± 20.64	76.44 ± 0.0

**Table 3:** Effect of metals on the activity of immobilized protocatechuate 3,4-dioxygenase from *Stenotrophomonas maltophilia* KB2. Data shown represent the average of three independent trials ± standard deviation.

Compound	Concentration, (mM)	Relative activity of free enzyme, % [6]	Relative activity of the enzyme immobilized in calcium alginate, %	Relative activity of the enzyme immobilized on glyoxyl agarose, %
None		100.0 ± 0.00	100.0 ± 0.00	100.0 ± 0.00

Co^2+^	1	74.34 ± 19.14	39.68 ± 8.98	61.65 ± 8.88
	2	70.58 ± 8.45	70.63 ±7.86	67.27 ± 0.00
	3	51.55 ± 4.70	97.62 ± 3.37	86.11 ± 9.82

Cu^2+^	1	41.81 ±4.07	30.16 ± 0.00	85.12 ± 0.00
	2	27.36 ± 2.61	28.57 ± 2.24	80.16 ± 5.61
	3	29.64 ± 0.63	22.22 ± 0.00	85.12 ± 0.00

Ni^2+^	1	78.65 ± 3.02	70.27 ± 0.00	69.58 ± 0.00
	2	88.34 ± 2.29	84.24 ± 3.84	44.63 ± 10.05
	3	83.85 ± 0.31	63.51 ± 11.47	59.67 ± 0.93

Mn^2+^	1	108.06 ± 8.19	50.40 ± 1.13	70.74 ± 0.70
	2	98.55 ± 5.08	20.00 ± 3.39	75.87 ± 6.76
	3	80.92 ± 5.43	29.60 ± 3.39	83.30 ± 2.10

Cd^2+^	1	69.33 ± 4.01	106.76 ± 7.64	84.70 ± 7.03
	2	63.85 ± 2.13	115.54 ± 8.60	112.62 ± 6.49
	3	60.20 ± 12.64	62.16 ± 3.82	93.12 ± 8.11

Al^3+^	1	89.99 ± 5.43	46.62 ± 10.51	87.76 ± 4.33
	2	68.39 ± 3.21	83.11 ± 0.96	78.97 ± 9.19
	3	33.50 ± 2.14	131.08 ± 11.47	91.20 ± 17.31

Zn^2+^	1	94.27 ± 7.21	27.20 ± 11.31	70.74 ± 0.70
	2	71.79 ± 5.17	11.20 ± 6.79	82.97 ± 3.97
	3	50.95 ± 2.76	4.80 ± 0.00	67.76 ± 9.58

Fe^3+^	1	38.93 ± 6.17	180.16 ± 19.08	61.50 ± 0.00
	2	31.17 ± 6.08	150.79 ± 9.00	56.03 ± 6.54
	3	22.76 ± 2.11	141.27 ± 6.73	54.38 ± 5.61

**Table 4:** Effect of chelators on the activity of immobilized protocatechuate 3,4-dioxygenase from *Stenotrophomonas maltophilia* KB2. Data shown represent the average of three independent trials ± standard deviation.

Compound	Concentration, (*μ*M)	Relative activity of free enzyme, %	Relative activity of the enzyme immobilized in calcium alginate, %	Relative activity of the enzyme immobilized on glyoxyl agarose, %
None		100.0 ± 0.00	100.0 ± 0.00	100.0 ± 0.00

EDTA	100	135.07 ± 1.86	113.13 ± 7.11	46.72 ± 27.85
	200	128.19 ± 23.46	97.54 ± 3.91	30.89 ± 6.55
	300	18.19 ± 0.12	116.92 ± 8.35	32.43 ± 3.28

2,2′-dipyridyl	100	119.78 ± 0.87	123.81 ± 6.58	57.91 ± 0.00
	200	99.30 ± 0.99	111.62 ± 3.55	30.12 ± 17.47
	300	87.62 ± 4.85	117.40 ±3.56	22.01 ± 12.56

Phenanthroline	100	97.78 ± 7.33	117.53 ± 7.64	101.93 ± 1.09
	150	81.46 ± 4.10	121.04 ± 5.87	60.62 ± 16.93
	200	69.69 ± 3.11	101.56 ± 6.76	21.62 ± 7.64
